# Molecular characterization of *Helicobacter pylori* clinical isolates from Eastern Saudi Arabia

**DOI:** 10.15537/smj.2022.43.10.20220355

**Published:** 2022-10

**Authors:** Khaled R. Alkharsah, Reem Y. Aljindan, Aisha M. Alamri, Amer I. Alomar, Abdulaziz A. Al-Quorain

**Affiliations:** *From the Department of Microbiology (Alkharsah, Aljindan), College of Medicine; from the Department of Clinical Laboratory Sciences (Alamri, Alomar), College of Applied Medical Sciences, Imam Abdulrahman Bin Faisal University, Dammam, and from the Department of Gastroenterology (Al-Quorain), King Fahd Hospital of the University, Alkhobar, Kingdom of Saudi Arabia.*

**Keywords:** *H. Pylori*, clarithromycin, resistance, Saudi Arabia, *23s rRNA*

## Abstract

**Objectives:**

To describe the frequency of *cytotoxin-associated gene A* (*CagA*) and *vacuolating cytotoxin A* (*VacA*) virulence genes and clarithromycin resistance-associated mutations among *Helicobacter pylori* (*H. pylori*) clinical isolates from Eastern Saudi Arabia.

**Methods:**

A cross-sectional study was carried out between July 2020 and June 2021 in a tertiary hospital in AL-Khobar, Saudi Arabia. A total of 34 *H. pylori* isolates were obtained from gastric biopsies of patients with dyspepsia. The existence of the virulence genes was studied by polymerase chain reaction and the gene fragment of the *23s ribosomal subunit* (*23s rRNA*) gene was sequenced.

**Results:**

All isolates harbored the *CagA* gene. Approximately 97.1% (33/34) isolates were positive using the *VacA* M primer and 91.2% (31/34) isolates were positive using the *VacA* S primer. The most frequent allelic combination was S2/M2/cag (60%), followed by S1/M2/cag (26.7%), S1/M1/cag (10%), and S2/M1/cag (3.3%). Approximately 6.5% isolates harbored the *A2142G* mutation and 29% isolates harbored the *A2143G* mutation. One isolate contained the mutation *T2182C*. The phylogenetic analysis showed that 58% isolates clustered with the regional and global isolates while the remaining 42% isolates seemed to be specifically circulating in Saudi Arabia. Most of the patients (73.5%) had already underwent a previous *H. pylori* eradication therapy.

**Conclusion:**

We showed that there is a regional variation in the frequency of the virulence genes among *H. pylori* isolates. Additionally, we showed the frequency of *23s rRNA* mutations related to clarithromycin resistance in Saudi Arabia.

***H**elicobacter pylori* (*H. pylori*) has been recognized since a long time as a significant pathogen for the stomach and duodenal tissues and the most common cause of gastric ulcers and gastric cancer.^1^ The main treatment regimen for *H. pylori* is a combination of clarithromycin (CLR), a proton pump inhibitor, and amoxicillin or metronidazole.^1^ However, the emergence of *H. pylori* strains that are resistant to CLR and other antibiotics and its implication in failure of treatment has warranted the need to discover novel treatment strategies.

Clarithromycin is a macrolide antibiotic which targets the *23s ribosomal subunit* (*23s rRNA*) and mutations within the V domain of the *23s rRNA* are mostly linked to macrolides resistance.^2^ Globally, studies have found that the pattern of resistance to clarithromycin, levofloxacin, and metronidazole in *H. pylori* isolates exceeded 15% in many parts of the world.^3^ Additionally epidemiological studies assessing the prevalence of clarithromycin resistance have indicated a resistance level that ranges from 5.5-30.8% with the highest trend in Asia (27.5%).^2^

Resistance to CLR was mainly attributed to point mutations in the gene *23s rRNA*.^4^ In addition, multidrug efflux pumps can also contribute to a conserved resistance to CLR in *H. pylori* strains.^5^ Moreover, point mutations in *rpl22* and *infB* genes have also been implicated in CLR resistance.^6^ In this context, CLR resistance is a determining factor in successful treatment and eradication of *H. pylori*; therefore, recent guidelines recommended that *H. pylori* treatment strategies should depend on the degree of CLR resistance.^7^ Bearing in mind the importance of CLR resistance in *H. pylori*, introduction of levofloxacin antibiotic to the treatment regimen resulted in the emergence of strains resistant to this antibiotic as well. Levofloxacin resistance is associated, at least in part, with mutations in the region determining quinolone resistance, which contains 2 genes encoding the gyrase enzyme isoforms A and B.^8,9^ This information on levofloxacin and CLR resistance was supported by a German study, which reported a 6.9% prevalence of CLR and a 14% prevalence of levofloxacin resistance in Germany.^10^ Hence, linking the bacterial phenotype to the genotype and the map of antimicrobial resistance could be significant to deriving better clinical decisions on the choice of the suitable antibiotic that could be more effective depending on bacterial strain genotype.

The *vacuolating cytotoxin A* (*VacA*) as well as *cytotoxin associated antigen A* (*CagA*) are thought to be the major *H. pylori* pathogenic genes.^11^ *Cytotoxin associated antigen A* gene is the principal protein encoded by cytotoxin associated gene pathogenicity island and has been linked to the virulent strains that have been found to be linked to peptic ulcer disease and gastric carcinoma. On the other hand, *VacA* is a common gene among isolates of *H. pylori*. However, some gene alleles were linked to chronic inflammation of gastric mucosa.^11^ *Helicobacter pylori* strains carrying the *VacA* gene may differ depending on the variation in *VacA* regions namely, s-, i-, m-, d-, and c-regions with *VacA* s1/m1 alleles are known to be among the most virulent *H. pylori* strains.^12^

The prevalence of *H. pylori* in Saudi Arabia ranges between 28-46% among adults with dyspepsia depending on the geographical location; the highest being in the southern region.^13-16^ Among children, the seroprevalence of *H. pylori* in Saudi Arabia was reported to be 40%.^17^ Therefore, understanding the pathogenesis of the circulating strains and antimicrobial resistance pattern, and their molecular background is crucial for proper treatment of *H. pyroli* infection.

Thus, the aim of the current study was to explore the genetic makeup of *H. Pylori* isolates obtained from patients in Saudi Arabia and to identify potential antibiotic resistance mutations to get insight on the most successful therapy combinations to eradicate this microorganism and attenuate its virulence-related pathogenicity.

## Methods

A cross-sectional study was carried out between July 2020 and June 2021 in a tertiary hospital in AL-Khobar, Saudi Arabia. Gastric biopsies from patients who underwent gastroduodenal endoscopy were received at the diagnostic Microbiology Laboratory at King Fahd Hospital of the University, Al-Khobar, Saudi Arabia, for the detection and isolation of *H. pylori*. No patient sample was specifically collected for the purpose of this project. Only isolated *H. pylori* strains were collected and used in the study. Patient’s clinical data were collected from the patient’s medical record and data analysis was carried out anonymously.

The inclusion criteria was any *H. pylori* strain isolated in culture from gastric biopsy and no exclusion criteria was carried out. *Helicobacter pylori* isolates were collected from 34 gastric biopsies from 17 males and 17 females. A total of 28 (82.4%) patients were from Saudi Arabia, 2 from Jordan, and one each from Yemen, Morocco, Eritrea, and Indonesia. The patient’s age ranged between 19-73 years (median 35 years and average 38 years).

The ethical approval for the study was obtained from the Institutional Review Board at Imam Abdulrahman Bin Faisal University, Dammam, Saudi Arabia, (approval number: IRB-2020-03-229).

Since no samples were directly obtained from the patients for the purpose of this study and only the isolated bacterial strains were used in the study, no consent form was required.

*Helicobacter pylori* clinical isolates were obtained from the diagnostic microbiology laboratory at the University hospital. The routine processing of gastric biopsies for isolation of *H. pylori* was carried out as follows: after homogenization of the biopsy sample in a manual tissue grinder containing 1 ml normal saline, 350 μl homogenate was inoculated on Colombia agar supplemented with 10% horse serum and Dent supplement (SPML, Riyadh, Saudi Arabia). Additionally, 350 μl of the grinded tissue were inoculated on 5% sheep blood agar and on chocolate agar (SPML, Riyadh, Saudi Arabia). All plates were incubated in microaerophilic condition using Oxoid CampyGen Gaspak (ThermoFisher Scientific, Hampshire, UK) for 4-10 days. Any bacterial growth on any of the plates was tested by Gram stain and other biochemical tests. A heavy inoculum of the identified *H. pylori* isolates was resuspended in 300 μl phosphate buffered saline and stored at -80°C for molecular characterization.

The QiaAmp DNA extraction kit (Qiagen, Hilden, Germany) was utilized for bacterial DNA extraction following the manufacturer’s instruction. The presence of major genes that correlate with the *H. pylori* virulence (*VacA* S, *VacA* M, and *CagA*) was studied by PCR using the primers shown in Table 1.^18,19^ Cycling protocols were employed as previously described.^18^ The visualization of the PCR product was carried out gel electrophoresis.

**Table 1:** - Primer sequences and amplicon size.

Primers	Sequences	Band sizes (bp)	References
*VacA* (s)-F	5’ATGGAAATACAACAAACACAC3’	s1: 259, s2: 286	18,19
*VacA* (s)-R	5’CTGCTTGAATGCGCCAAAC3’		
*VacA* (m)-F	5’CAATCTGTCCAATCAAGCGAG3’	m1: 570, m2: 642	
*VacA* (m)-R	5’GCGTCTAAATAATTCCAAGG3’		
*CagA*-F	5’AATACACCAACGCCTCCA3’	400	
*CagA*-R	5’TTGTTGCCGCTTTTGCTCTC3’		
23s-F	ATGAATGGCGTAACGAGATG	361	20
23s-R	GGAAATCGCAAGTTGAGTGT		

*VacA:* vacuolating cytotoxin A, *CagA:* cytotoxin associated antigen A

The fragment of the *23s rRNA* gene that contains the CLR resistance associated mutations was amplified by PCR employing the primers enumerated in Table 1.^20^ The amplified PCR fragment was excised from the agarose gel and purified. The purified DNA sample was sequenced using the big dye terminator mix (Thermo Fisher Scientific, Massachusetts, USA) in both forward and reverse directions.

Sequences were cleaned and a consensus sequence was obtained from the forward and reverse sequences for each isolate. The sequences were aligned using ClustalW multiple alignment function in the software BioEdit, version 7.0.4.1. The phylogenetic tree was generated via the Neighbor-Joining method.

**Table 2:** - Demographic and clinical data of the patients infected with *Helicobacter pylori*.

Variables	Male (n=17)	Female (n=17)
***Age (years)***		
19-30 31-40 41-50 51-60 >60	7 (53.8) 4 (50.0) 2 (40.0) 3 (42.9) 1 (100)	6 (46.1) 4 (50.0) 3 (60.0) 4 (57.1) 0 (0.0)
***Nationality***		
Saudi Non-Saudi	14 (50.0) 3 (50.0)	14 (50.0) 3 (50.0)
***Endoscopic findings (gastritis)***		
Mild Moderate Severe Nodular Erosive	5 (41.7) 4 (66.7) 2 (100) 2 (66.7) 3 (75.0)	7 (58.3) 2 (33.3) 0 (0.0) 1 (33.3) 1 (25.0)
***Prior eradication therapy***		
Unknown Once Twice	4 (50.0) 13 (52.0) 0 (0.0)	4 (50.0) 12 (48.0) 1 (100)

Values are presented as number and precentage (%).

All sequences from the bacterial isolates were deposited in the The National Center for Biotechnology Information data bank (accession numbers: MZ350604-MZ350633).

### Statistical analysis

Data were tabulated in Microsoft Excel spreadsheets. All frequencies and percentages were calculated in Microsoft Excel. Statistical associations were calculated using the OpenEpi website and employing the 2x2 tables. The mid-*p* exact test was used to evaluate the association with a significant *p*-value of <0.05.

## Results

Patients’ characteristics are shown in Table 2. There was no statistically significant difference in the rate of infection between females and males from different age groups (*p*=0.786).

Endoscopic findings showed that most patients have normal mucosa with mild to severe gastritis or erosive gastritis with or without nodules (Table 2). Most of the patients (25/34, 73.5%) had already underwent a previous *H. pylori* eradication therapy (Table 2).

**Figure 1:**
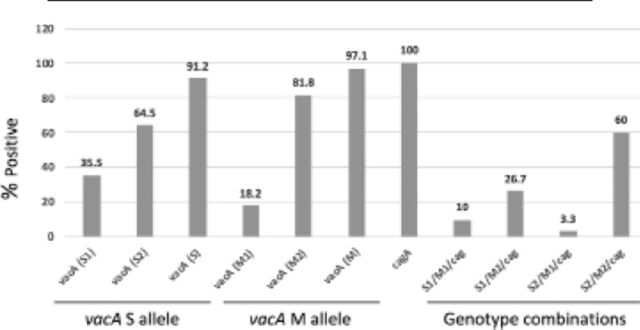
- Percentage of positive virulence genes and genotype combinations in *Helicobacter pylori* isolates. The *VacA* S1 and S2 values are calculated from the *VacA* S total positive.^31^ The *VacA* M1 and M2 values are calculated from the *VacA* M total positive.^33^ The *VacA* S value is calculated from the total isolates.^34^ The *VacA* M value is calculated from the total isolates.^34^ The genotypes combinations values are calculated out of 30 isolates. *VacA*: *vacuolating cytotoxin A*

All of the isolates were positive for the *CagA* gene. Approximately 97.1% (33/34) isolates were found to be positive using *VacA* M primers (6 M1 and 27 M2) and 91.2% (31/34) isolates were found to be positive using *VacA* S primers (11 S1 and 20 S2; Figure 1). The most frequent allelic combinations between *VacA* variants and the *CagA* were S2/M2/Cag (18/30), followed by S1/M2/Cag (8/30), S1/M1/Cag (3/30), and S2/M1/Cag (1/30). The S2 variant was more represented with the moderate and nodular gastritis (5/1) than the S1 variant (3/0); however, this representation was statistically insignificant.

**Figure 2:**
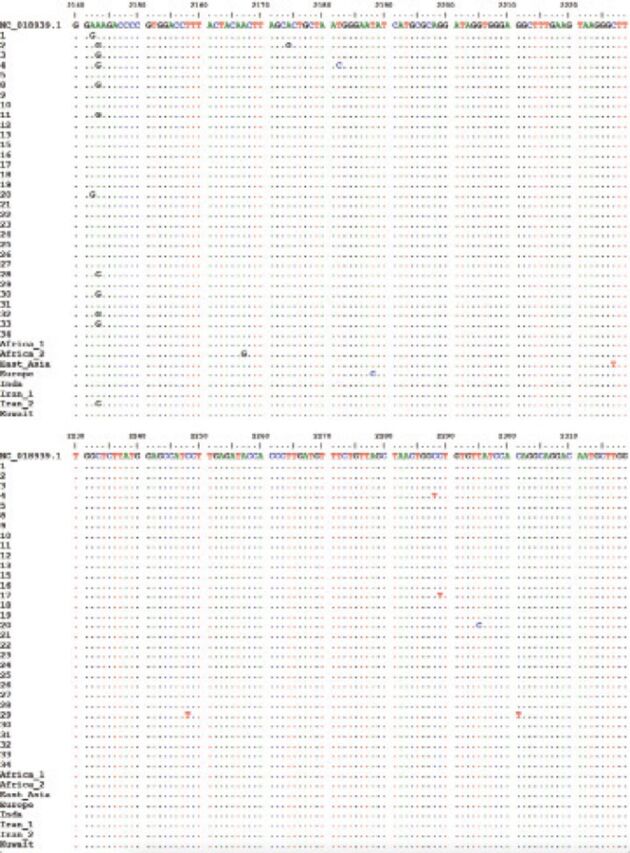
- Sequence alignment of the *23s rRNA* gene fragment.

**Figure 3:**
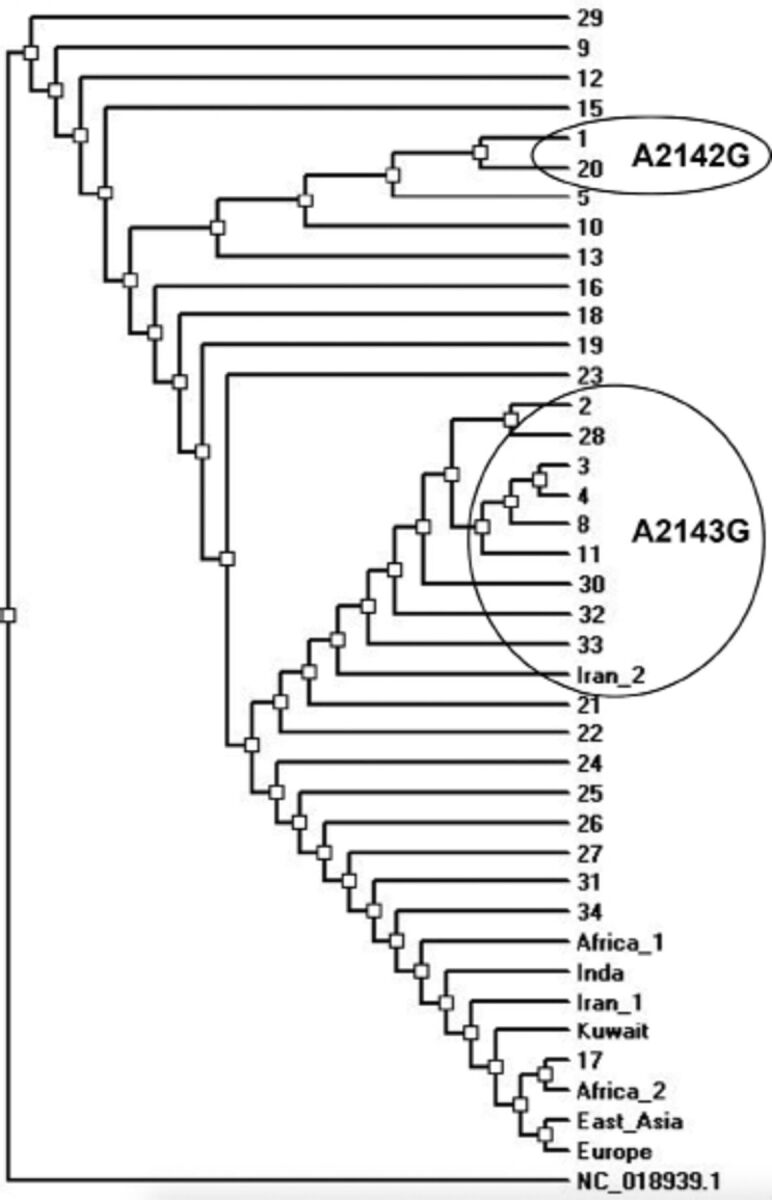
- Phylogenetic tree of the *Helicobacter pylori* isolates from Saudi Arabia and other regional and global isolates based on the *23s rRNA* gene fragment. The circles indicate the isolates with the mentioned mutations.

Sequence analysis for the *23s rRNA* gene fragment was obtained from 31 isolates. Sequence analysis showed that 2 (6.5%) isolates harbored the A2142G mutation and 9 (29%) isolates harbored the A2143G mutation. One isolate contained the mutation T2182C while the rest of isolates had single scattered mutations (Figure 2).

The phylogenetic tree of the local and global isolates based on the *23s rRNA* gene fragment is shown in Figure 3. Approximately 58% of the isolates clustered with the regional and global isolates. The isolates with the *A2143G* mutation clustered together with a similar isolate reported in Iran. The remaining 42% of the isolates including those harboring the *A2142G* mutation seemed to be specifically circulating in Saudi Arabia (Figure 3).

## Discussion

The current study describes the prevalence of the *VacA* and *CagA* virulence genes and mutations related to CLR resistance in *H. pylori* clinical isolates from the Eastern province of Saudi Arabia.

All *H. pylori* strains in this study were positive for *CagA*, which is uncommon to other strains from the same region.^11,21,22^ The *CagA* protein is an important virulence protein, which is introduced into the host cell through a secretion system of type IV.^23^ It is responsible for enhancing inflammation in gastric mucosa by inducing DNA damage and oxidative stress leading to increased risk of gastric cancer.^24^ This may explain the presence of mild to severe gastritis in the majority of the patients in the study. Horiuchi et al^25^ suggested a correlation between *CagA* positive strain and recurrent *H. pylori* infection. Indeed, approximately 75% of the patients in the study did undergo a previous eradication therapy. However, a reinfection scenario cannot be confirmed nor a failure of the eradication therapy due to incompliance with the treatment cannot be precluded at this stage.

*Vacuolating cytotoxin A* is another important virulence protein of *H. pylori* encoded by the gene *VacA*. In addition to induction of vacuolation inside host cells, *VacA* induces inflammation, inhibits T-cell activation, and triggers cellular apoptosis via the mitochondrial intrinsic pathway.^26^ *Helicobacter pylori* strains expressing *VacA* show an increased tendency to cause peptic ulceration and gastric cancer.^26^ *Vacuolating cytotoxin A* seropositive individuals were shown to be at higher risk of developing gastric cancer that occurred mostly in association with the S1/M1 allele.^27^ The *VacA* allele s1bm1 was prevalent in more than half of the isolates in a study from North Arizona.^28^ European strains of *H. pylori* harbor multiple *VacA* allelic types with or without the *Cag* pathogenicity island, while the East Asian S1 or S2 strains that lacks the *Cag* pathogenicity island usually do not exist.^29^

The most common *VacA* allele in our study was the S2/M2 allele. This allele did not show in vitro *Vac* activity, which might explain the mild form of the disease in our patients.^30^ It was reported previously that the S1/M1 allele is responsible for higher level of virulence, compared to other alleles, followed by the S1/M2 allele.^26^ These alleles were present in only 10% and 26% of our isolates. However, they were not associated with increased severe clinical picture. Different studies from Saudi Arabia reported different prevalence of the *VacA* alleles. The S1/M1 allele is the most prevalent in the southern region of Saudi Arabia, while S1/M2 allele is more prevalent in the middle and the western regions of the country.^11,31,32^ The most frequent allele in our study was S2/M2, followed by S1/M2, which indicated a regional variation. The S2/M1 allele was only found in 3% of our isolates and was not previously reported in Saudi Arabia.^11,22,33^

As CLR is the first line therapy for *H. pylori* related infections, understanding the mechanisms underlying the development of CLR resistance is crucial.^34^ The overall resistance to CLR in Saudi Arabia was reported to range between 23-40%.^35,36^ Several studies have linked the *23s rRNA* gene mutations of *H. pylori*, including A2142G, A2143G, or T2182C, among others, to CLR resistance.^37^ Nonetheless, multiple studies reported a discrepancy between the phenotypic resistance and mutation detection.^38^ A study from Iraq showed that the clarithromycin related mutations are very common (60.5% for A2144G and 50% for A2143).^39^ In a study from China, 82.8% of the clarithromycin resistant isolates contained the mutation A2143G and 89.7% contained the mutation T2182C, while 45.5% of the clarithromycin sensitive isolates contained the A2143G mutation.^40^

Approximately 35.5% (11/31) of the isolates in our study had either one of the 3 mutations mentioned above. However, 73.5% of the study population had a previous eradication treatment. Whether this is attributed to one of the detected mutations cannot be confirmed due to the lack of phenotypic antimicrobial susceptibility for the isolates. Additionally, the possibility of reinfection and incompliance with the treatment cannot be precluded.

The phylogenetic tree showed a nice clustering of genotypes with other regional and global strains and reflects the multinational atmosphere in the country. Furthermore, a group of strains seems to be circulating in Saudi Arabia but this could be attributed to the short sequence included in the analysis.

### Study limitations

The little number of isolates and the lack of antimicrobial susceptibility testing data, which preclude a firm conclusion regarding the association of the CLR mutations with phenotypic resistance.

In conclusion, our study showed that there is a regional variation in the prevalence of virulence genes among *H. pylori* isolates. Additionally, we showed that the prevalence of the *23s rRNA* mutations-related to CLR resistance is not high among the isolates, despite the fact that most of the patients did already receive eradication treatment. However, as above mentioned, phenotypic clarithromycin resistance testing is needed to confirm this conclusion. Furthermore, we presented the phylogenetic relatedness of the *H. pylori* isolates and showed that most isolates cluster with isolates from other countries, while some isolates are circulating in Saudi Arabia.
